# Association of Complete Metabolic Response on Post-Neoadjuvant 18F-FDG PET/CT with Complete Cytoreduction in Advanced High-Grade Serous Tubo-Ovarian Carcinoma: A Retrospective Single-Center Cohort Study

**DOI:** 10.3390/diagnostics16193164

**Published:** 2026-09-29

**Authors:** Basak Ozge Kayan, Ipek Ozturk, Yahya Ozgun Oner, Burcu Esen Akkas, Meryem Kaya, Nilufer Bulut, Semra Altinay, Gozde Sahin, Isik Sozen, Ilkbal Temel Yuksel

**Affiliations:** 1 Department of Gynecologic Oncology, Basaksehir Cam and Sakura City Hospital, 34480 Istanbul, Turkey; yahyaozgunoner@gmail.com (Y.O.O.); sahin.gozde1983@gmail.com (G.S.); isiksozen@gmail.com (I.S.); drilkbaltemel@gmail.com (I.T.Y.); 2 Department of Nuclear Medicine, Basaksehir Cam and Sakura City Hospital, 34480 Istanbul, Turkey; ipekkerimel@gmail.com (I.O.); burcuesen@yahoo.com (B.E.A.); bmeryemkaya@yahoo.com (M.K.); 3 Department of Medical Oncology, Basaksehir Cam and Sakura City Hospital, 34480 Istanbul, Turkey; ferlut@gmail.com; 4 Department of Pathology, Basaksehir Cam and Sakura City Hospital, 34480 Istanbul, Turkey; vuralsemra@hotmail.com

**Keywords:** high-grade serous ovarian carcinoma, 18F-FDG PET/CT, complete metabolic response, neoadjuvant chemotherapy, interval debulking surgery, complete cytoreduction

## Abstract

**Background/Objectives:** To evaluate whether complete metabolic response (CMR) on 18F-FDG PET/CT after neoadjuvant chemotherapy (NACT) is associated with complete cytoreduction at interval debulking surgery (IDS), progression-free survival (PFS), and overall survival (OS) in patients with advanced high-grade serous tubo-ovarian carcinoma. **Methods:** This retrospective single-center cohort included 87 patients with FIGO stage III–IV high-grade serous carcinoma undergoing 18F-FDG PET/CT before and after NACT. Metabolic response was classified as CMR or non-CMR using criteria adapted from PERCIST. The primary outcome, complete cytoreduction (R0), was assessed using multivariable logistic regression adjusted for age, FIGO stage, and number of NACT cycles. PFS and OS were analyzed from the interval debulking surgery (IDS) landmark using the Kaplan–Meier method, and PFS was further evaluated with Cox regression. **Results:** CMR was observed in 34 patients (39.1%). Complete cytoreduction was achieved in 62 (71.3%) and was more frequent with CMR (85.3% vs. 62.3%; *p* = 0.025). CMR was independently associated with R0 (adjusted OR 3.56, 95% CI 1.18–10.75; *p* = 0.024; sensitivity 46.8%, specificity 80.0%, PPV 85.3%). In the IDS-landmark analysis, median PFS was longer in the CMR group (12.9 vs. 9.1 months; *p* = 0.027), but this association was attenuated and no longer significant after adjustment for R0. OS did not differ significantly between the groups (*p* = 0.259). **Conclusions:** Post-NACT CMR was independently associated with complete cytoreduction and may support preoperative risk stratification. However, given its limited sensitivity, CMR should not be used as the sole determinant for IDS.

## 1. Introduction

Ovarian cancer has one of the highest mortality-to-incidence ratios among gynecologic malignancies, largely because most patients are diagnosed at an advanced stage [1]. In advanced high-grade serous tubo-ovarian carcinoma, the completeness of cytoreduction is the strongest modifiable prognostic factor, and achieving no macroscopic residual disease (R0) is the principal surgical goal [2,3]. When upfront optimal cytoreductive surgery is not feasible, neoadjuvant chemotherapy (NACT) followed by interval debulking surgery (IDS) is an accepted treatment strategy [4]. However, because the survival benefit of IDS depends largely on achieving complete cytoreduction, identifying patients most likely to achieve this outcome before surgery remains a major clinical challenge [2,3,4].

Accurate assessment of response to NACT is therefore essential when selecting candidates for IDS. Contrast-enhanced computed tomography (CT), interpreted by size-based Response Evaluation Criteria in Solid Tumors (RECIST), reflects morphological change and correlates only modestly with the viable tumor that remains after chemotherapy [5,6]. By contrast, 18F-fluorodeoxyglucose positron emission tomography/computed tomography (18F-FDG PET/CT) images glucose metabolism and can distinguish viable tumor from treatment-induced fibrosis; a complete metabolic response (CMR) after NACT has been associated with pathological response and clinical outcomes in other solid tumors, including breast and rectal cancer [7,8]. Because morphologic shrinkage on CT may lag behind, or overestimate, the amount of viable tumor remaining after chemotherapy, a functional marker of residual disease could provide a more direct estimate of the likelihood of complete resection. However, whether the metabolic response after NACT is associated with complete cytoreduction in ovarian cancer remains uncertain.

Only a few studies have evaluated post-NACT PET/CT in ovarian cancer, and most have focused primarily on survival rather than surgical outcomes [6,9]. The reported relationship between metabolic response and complete cytoreduction is contradictory: some series found more frequent complete cytoreduction among patients who achieved CMR [10], whereas others found no difference in the R0 rate between the CMR and non-CMR groups [5]. Furthermore, evidence remains limited regarding whether CMR is associated with complete cytoreduction in a histologically uniform, high-grade serous cohort. High-grade serous carcinoma is the most common and most chemosensitive subtype, and restricting the analysis to this histologic subtype reduces biological heterogeneity that may confound the relationship between metabolic response and surgical outcome. This uncertainty is clinically relevant because a reliable preoperative marker of resectability could improve patient selection for IDS.

The aim of this study was to evaluate whether CMR on post-NACT 18F-FDG PET/CT was associated with complete cytoreduction at IDS in patients with advanced high-grade serous tubo-ovarian carcinoma. Secondary analyses examined the associations between CMR and the Chemotherapy Response Score (CRS), progression-free survival (PFS), and overall survival (OS).

## 2. Materials and Methods

### 2.1. Study Design

This retrospective single-center cohort study was conducted at the Department of Gynecologic Oncology, Başakşehir Çam and Sakura City Hospital, Istanbul, Turkey. Consecutive patients treated between December 2022 and December 2025 were screened for eligibility.

### 2.2. Ethical Approval

The study was approved by the Scientific Research Ethics Committee No. 1 of Başakşehir Çam and Sakura City Hospital (decision no. 68, dated 25 February 2026; official letter no. E-96317027-514.10-306604610, dated 4 March 2026) and was conducted in accordance with the Declaration of Helsinki. At the time of admission, in accordance with the standard practice of our training and research hospital, patients provide general written consent permitting the use of their data, without personal identifying information, for scientific research. Study-specific informed consent for the present retrospective analysis was not separately obtained, and no additional examinations or interventions were performed for research purposes.

### 2.3. Data Sources and Participants

We retrospectively studied consecutive patients with advanced high-grade serous tubo-ovarian carcinoma (FIGO stage III–IV) treated at the Department of Gynecologic Oncology, Başakşehir Çam and Sakura City Hospital, between December 2022 and December 2025. Patients aged 18–80 years were eligible if they had received at least three cycles of platinum-based NACT, had undergone 18F-FDG PET/CT both before treatment and after completion of NACT (prior to IDS), had undergone interval debulking surgery, and had complete surgical and follow-up data. Patients were excluded if they had FIGO stage I–II disease, a non-high-grade serous histology, ovarian metastases from a primary malignancy outside the ovary, fallopian tube, or peritoneum, previous incomplete cytoreduction before NACT, inadequate PET/CT image quality, unavailable residual-disease status, or incomplete follow-up.

Demographic, clinical, treatment, imaging, surgical, and pathological data were extracted from the electronic medical records and the hospital pathology and operative databases. The collected variables comprised demographic characteristics (age, body mass index, menopausal status), tumor characteristics (FIGO stage and pre-NACT serum CA-125), treatment data (NACT regimen, number of cycles, and post-NACT serum CA-125), imaging data (pre- and post-NACT 18F-FDG PET/CT findings and the derived metabolic response), surgical and pathological data (extent of residual disease at IDS and the omental chemotherapy response score [CRS]), and follow-up data (disease recurrence and vital status). The analyzed variables were the primary exposure—post-NACT metabolic response (CMR vs. non-CMR)—and the primary outcome—complete cytoreduction (R0) at IDS; the secondary outcomes were CRS 3, PFS, and OS.

**Figure 1:**
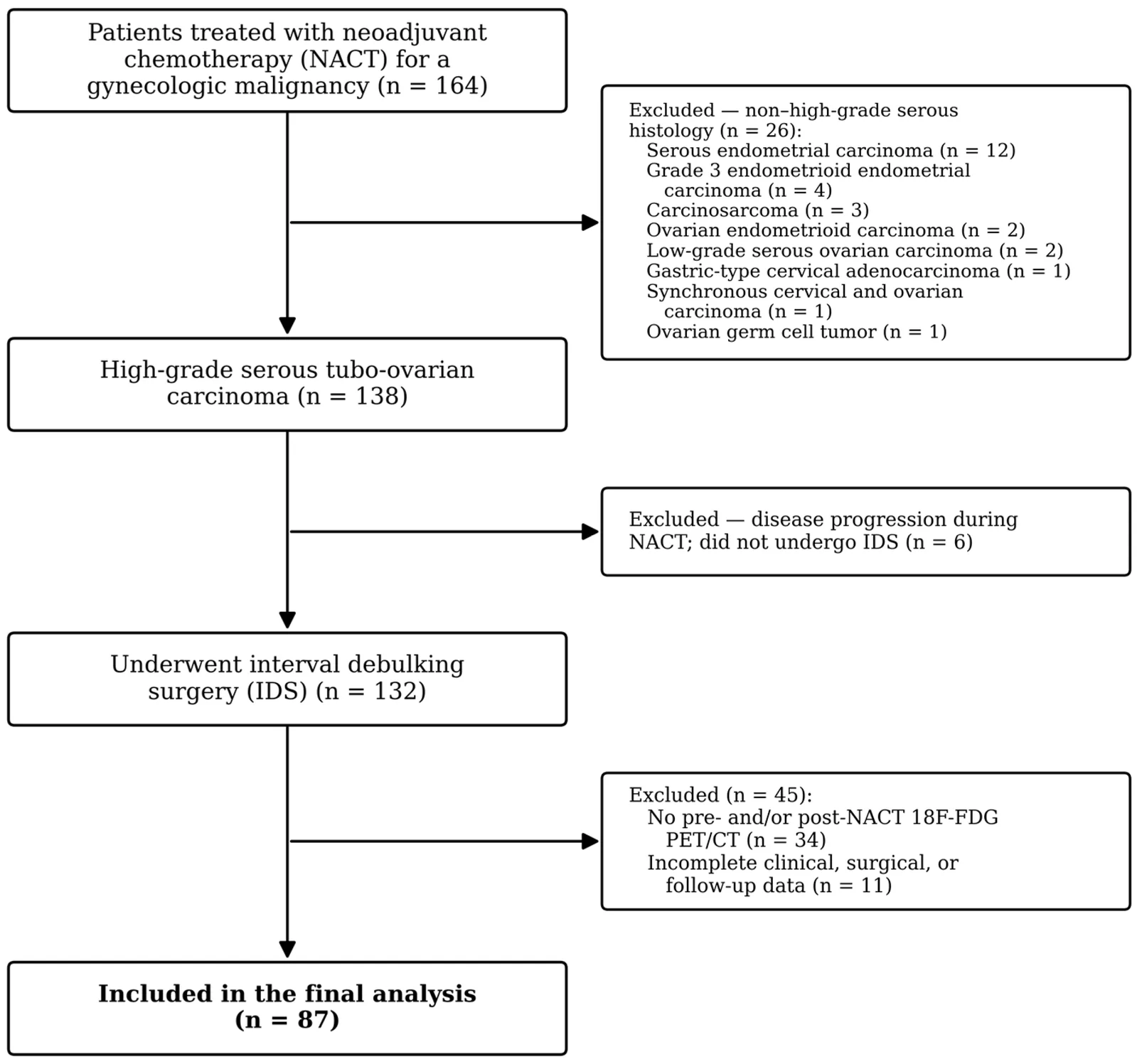
Flow diagram of patient selection and study cohort assembly. NACT, neoadjuvant chemotherapy; IDS, interval debulking surgery; PET/CT, positron emission tomography/computed tomography.

All consecutive patients who met the eligibility criteria during the study period were included; analyses were based on complete cases, without imputation of missing data. The patient-selection process and exclusions are summarized in Figure 1.

### 2.4. Working Protocol

***Diagnostic Confirmation and Neoadjuvant Treatment.*** The diagnosis of high-grade serous carcinoma was established before NACT by cytological or histological confirmation. Cytological confirmation was based on cytology of fluid obtained by paracentesis or thoracentesis, or of cells obtained by fine-needle aspiration biopsy; histological confirmation was based on core-needle (tru-cut) biopsy of an accessible suspected tumor site. When these cytological or histological investigations were non-diagnostic, diagnostic laparoscopy was performed to obtain tissue for diagnosis. All specimens were evaluated in the pathology department of our institution. The standard regimen consisted of intravenous carboplatin (area under the curve 5 mg·min/mL) and paclitaxel (175 mg/m^2^) every 21 days; two patients also received bevacizumab during the neoadjuvant treatment period. The number of cycles (three, four, or more) was determined by the treating medical oncology team according to treatment response and tolerability, and both the number of cycles and the intervals from completion of NACT to imaging and to surgery varied between patients.

***PET/CT Acquisition and Metabolic-Response Assessment.*** Pre- and post-NACT 18F-FDG PET/CT examinations were performed using an Ingenuity TF 64 PET/CT scanner (Philips Medical Systems, Cleveland, OH, USA). Patients fasted for at least 8 h before intravenous administration of 18F-FDG, and blood glucose was confirmed to be below 150 mg/dL before tracer injection. A dose of 0.10–0.14 mCi/kg (3.7–5.2 MBq/kg) was administered intravenously, and PET acquisition was initiated approximately 60 min later. A low-dose CT scan (120 kV, 113 mAs, 4 mm slice thickness) extending from the vertex to the mid-thigh was obtained for attenuation correction and anatomical localization, followed by PET emission imaging over the same range; images were reconstructed with the institutional standard iterative reconstruction protocol and CT-based attenuation correction. The post-treatment examination was performed approximately 2–4 weeks after completion of the final NACT cycle.

For the purposes of this study, all post-NACT PET/CT examinations were retrospectively re-evaluated rather than relying on the original clinical reports. Two experienced nuclear medicine physicians, each with more than 10 years of experience in oncological PET/CT interpretation and blinded to the surgical and histopathological findings and to each other’s assessments, independently reviewed the baseline and post-treatment examinations side by side. Discordant classifications were resolved by consensus, and the consensus classification was used in all analyses. Metabolic response was assessed using criteria adapted from the PET Response Criteria in Solid Tumors (PERCIST) [11,12]; because formal SULpeak quantification was not performed, response was assessed visually against the physiological reference of mean hepatic activity measured within uninvolved normal liver parenchyma (liver SUVmean). This retrospective CMR/non-CMR classification was performed for the purposes of the present study only and was not available to the multidisciplinary tumor board or to the operating surgeons, who relied on the routine clinical PET/CT report.

**Figure 2:**
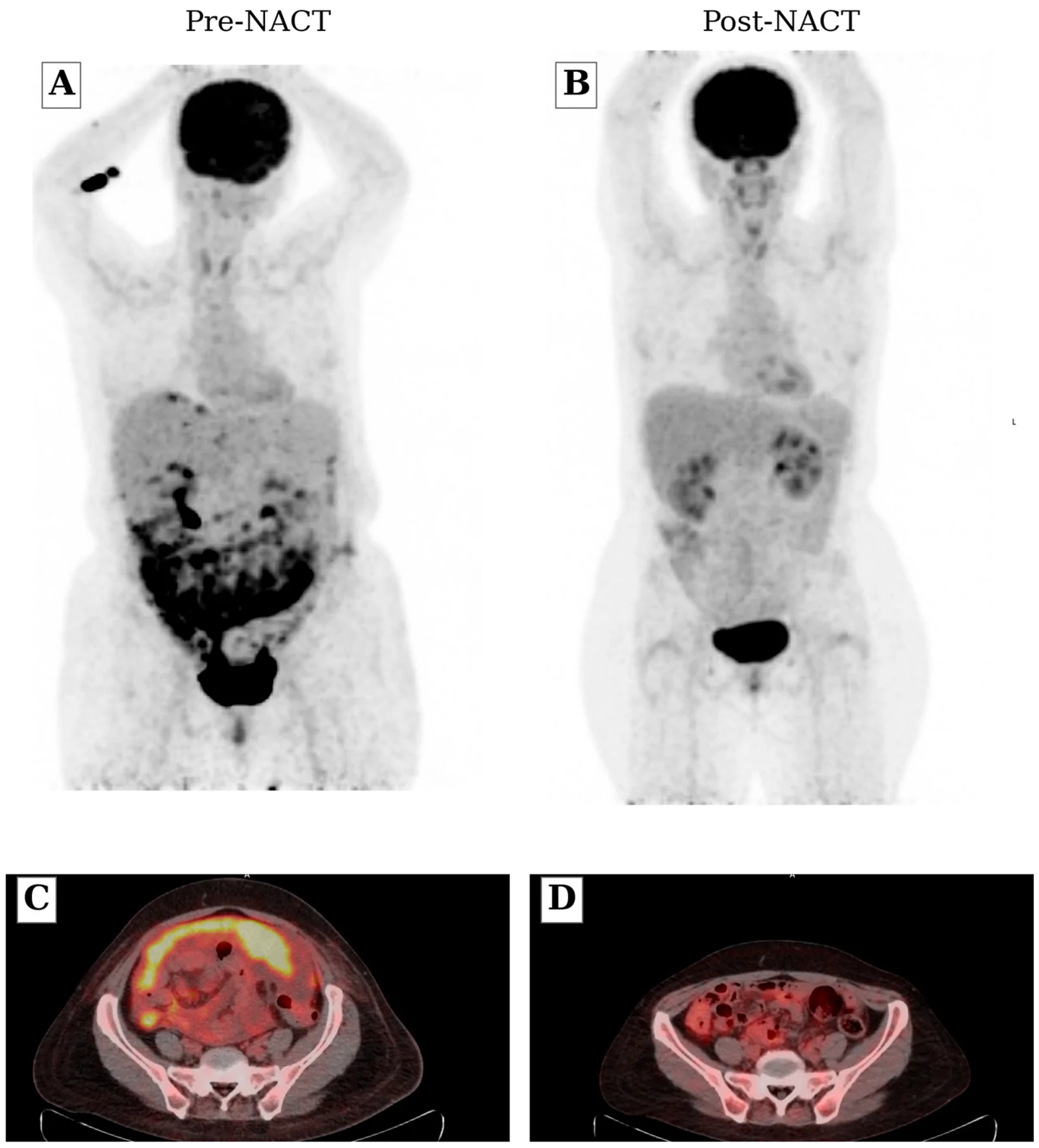
Representative example of a complete metabolic response (CMR) on post-neoadjuvant 18F-FDG PET/CT. Maximum-intensity-projection images before (**A**) and after (**B**) neoadjuvant chemotherapy (NACT) and fused axial PET/CT images before (**C**) and after (**D**) NACT in a patient classified as CMR, showing resolution of the previously pathological FDG uptake, with only physiological activity remaining. NACT, neoadjuvant chemotherapy.

**Figure 3:**
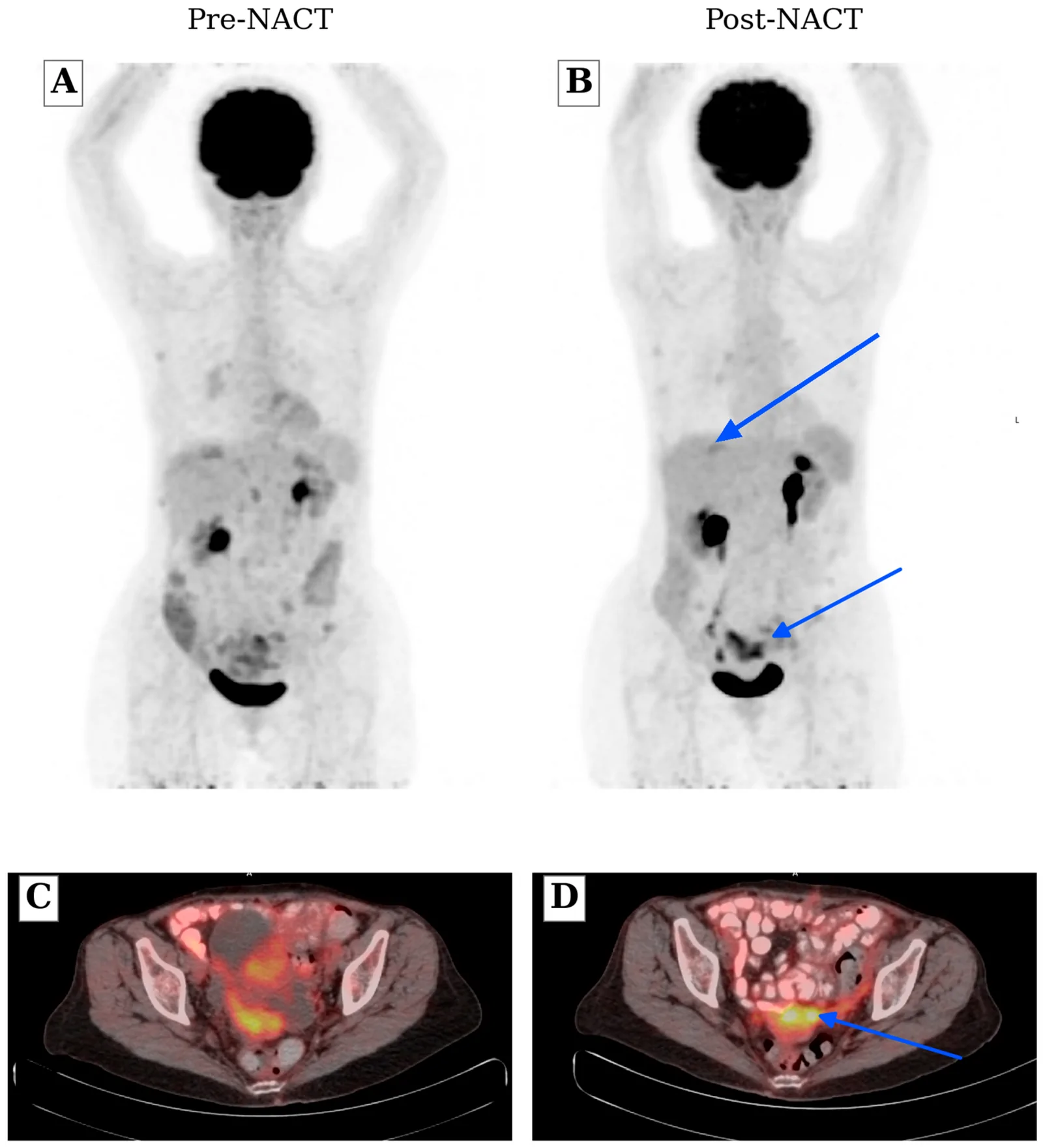
Representative example of a non-complete metabolic response (non-CMR) on post-neoadjuvant 18F-FDG PET/CT. Maximum-intensity-projection images before (**A**) and after (**B**) NACT and fused axial PET/CT images before (**C**) and after (**D**) NACT in a patient classified as non-CMR; the arrows (**B**,**D**) indicate residual FDG-avid disease after NACT.

Metabolic response was assessed at the patient level across all previously involved disease sites—the primary ovarian lesion, peritoneal implants, lymph nodes, and distant metastatic sites when present. A CMR was defined as complete resolution of abnormal 18F-FDG uptake in all previously hypermetabolic lesions to a level not exceeding normal hepatic activity and indistinguishable from surrounding background, with no new FDG-avid lesions; residual structural abnormalities without abnormal FDG uptake did not preclude classification as CMR (Figure 2). Patients who did not meet the CMR criteria were classified as non-CMR and analyzed as a single group (Figure 3).

***Surgical Decision-Making and Interval Debulking Surgery.*** The decision to proceed to IDS was made by a multidisciplinary tumor board on the basis of clinical, laboratory, and imaging findings, including the routine clinical post-NACT PET/CT report; operative planning was not blinded to these routine clinical PET/CT findings. Surgery was performed approximately 3–6 weeks after the final NACT cycle by gynecologic oncology surgeons aiming for complete macroscopic cytoreduction, and additional procedures were performed as required to achieve this goal. Residual disease was categorized from the operative reports as no macroscopic residual disease (R0), residual disease < 1 cm, or residual disease ≥ 1 cm, and complete cytoreduction was defined as R0. Pathological response was scored on omental specimens using the three-tier chemotherapy response score (CRS) of Böhm et al. [13], with CRS 3 denoting a complete or near-complete response.

### 2.5. Outcomes

The primary outcome was R0 at IDS. Secondary outcomes were CRS 3, PFS, and OS. PFS was defined as the interval from the date of IDS to the first documented progression, recurrence, or death from any cause; no patient died before progression, and patients without an event were censored at the last follow-up. OS was defined as the interval from the date of IDS to death from any cause, with surviving patients censored at the last follow-up.

### 2.6. Statistical Analysis

Continuous variables were summarized as the median with interquartile range and compared with the Mann–Whitney U test; categorical variables were summarized as frequencies and percentages and compared with the chi-square or Fisher exact test. Inter-observer agreement between the two independent readers before consensus was assessed with Cohen’s κ (asymptotic 95% confidence interval). The sensitivity, specificity, and positive and negative predictive values of CMR for complete cytoreduction were calculated with 95% confidence intervals using the Wilson method.

Factors associated with complete cytoreduction were assessed by logistic regression; the primary multivariable model included CMR, age, FIGO stage, and number of NACT cycles. Because post-NACT CA-125 may reflect the treatment response itself, it was added only in a sensitivity model after natural-logarithmic transformation, and a further sensitivity analysis modeled the number of NACT cycles as a categorical variable (reference, three cycles). Logistic regression results are reported as odds ratios with 95% confidence intervals.

Survival analyses used the date of IDS as the time origin (landmark analysis). Median follow-up was estimated using the reverse Kaplan–Meier method. PFS and OS were estimated with the Kaplan–Meier method and compared with the log-rank test; confidence intervals for Kaplan–Meier survival probabilities were calculated using the log-log transformation. For PFS, Cox proportional hazards regression was performed with two nested models: Model A included CMR, age, FIGO stage, and number of NACT cycles, and Model B additionally included complete cytoreduction (R0); the proportional-hazards assumption was assessed with scaled Schoenfeld residuals, and tied event times were handled using the Breslow method. Because the number of deaths was limited, no multivariable Cox model was fitted for OS, in order to avoid overfitting. Cox regression results are reported as hazard ratios with 95% confidence intervals.

No formal sample size calculation was performed. Descriptive statistics, group comparisons, logistic regression, and diagnostic performance measures (with Wilson score 95% confidence intervals) were computed in Python 3.11 (statsmodels 0.15.0, SciPy 1.17.1, NumPy, and pandas); the landmark Kaplan–Meier, log-rank, and Cox proportional hazards analyses (including the proportional-hazards assessment with scaled Schoenfeld residuals) and the reverse Kaplan–Meier estimation of median follow-up were performed in R version 4.3.3 (survival package, version 3.5.8). Cohen’s κ and its asymptotic 95% confidence interval were computed in Python using statsmodels. Study data were maintained in IBM SPSS Statistics version 31. A two-sided *p* < 0.05 indicated statistical significance.

## 3. Results

Of 164 patients who received NACT for a gynecologic malignancy during the study period, 26 were excluded because of non-high-grade serous histology, 6 because of disease progression that precluded IDS, and 45 because of missing pre- and/or post-NACT PET/CT or incomplete clinical data; a total of 87 patients with advanced high-grade serous tubo-ovarian carcinoma comprised the final cohort (Figure 1). CMR on post-NACT 18F-FDG PET/CT was observed in 34 patients (39.1%), whereas 53 (60.9%) had non-CMR. Baseline demographic and clinicopathological characteristics were similar between the groups, except for a lower median post-NACT CA-125 level in the CMR group (22.2 vs. 46.6 U/mL; *p* = 0.005) (Table 1). The post-NACT PET/CT was performed a median of 20 days (IQR 19–22) after the final NACT cycle, IDS a median of 34 days (IQR 31–37) after the final cycle, and the interval between PET/CT and IDS was a median of 13 days (IQR 10–17). Inter-observer agreement between the two readers before consensus was moderate (Cohen’s κ = 0.58, 95% CI 0.40–0.75; 79.3% agreement). Interval debulking surgery comprised peritoneal and retroperitoneal procedures (total abdominal hysterectomy, bilateral salpingo-oophorectomy, omentectomy, and dissection of suspicious or palpable pelvic and/or para-aortic lymph nodes rather than systematic lymphadenectomy) in 76 patients (87.4%) and peritoneal procedures in 11 (12.6%); additional surgical procedures were performed in 19 patients (colon resection in 7, diaphragmatic resection or stripping in 3, small-bowel resection in 2, splenectomy in 1, and multiple resections in 6); these were mutually exclusive, patient-level categories.

**Table 1:** Clinical and treatment characteristics according to metabolic response.

Characteristic	CMR (*n* = 34)	Non-CMR (*n* = 53)	*p*
Age, years, median (IQR)	63.0 (54.5–67.0)	61.0 (55.0–70.0)	0.927
BMI, kg/m^2^, median (IQR)	26.2 (23.2–31.2)	27.3 (25.0–30.1)	0.712
Postmenopausal, *n* (%)	26 (76.5)	45 (84.9)	0.322
FIGO stage, *n* (%)			0.831
III	11 (32.4)	16 (30.2)	
IV	23 (67.6)	37 (69.8)	
NACT cycles, *n* (%)			0.450
3	7 (20.6)	12 (22.6)	
4	16 (47.1)	18 (34.0)	
>4	11 (32.4)	23 (43.4)	
Carboplatin–paclitaxel, *n* (%)	34 (100)	53 (100)	—
Baseline CA-125, U/mL, median (IQR)	1163 (535–2458)	712 (274–1965)	0.150
Post-NACT CA-125, U/mL, median (IQR)	22.2 (10.0–45.4)	46.6 (18.5–158.0)	0.005

**Figure 4:**
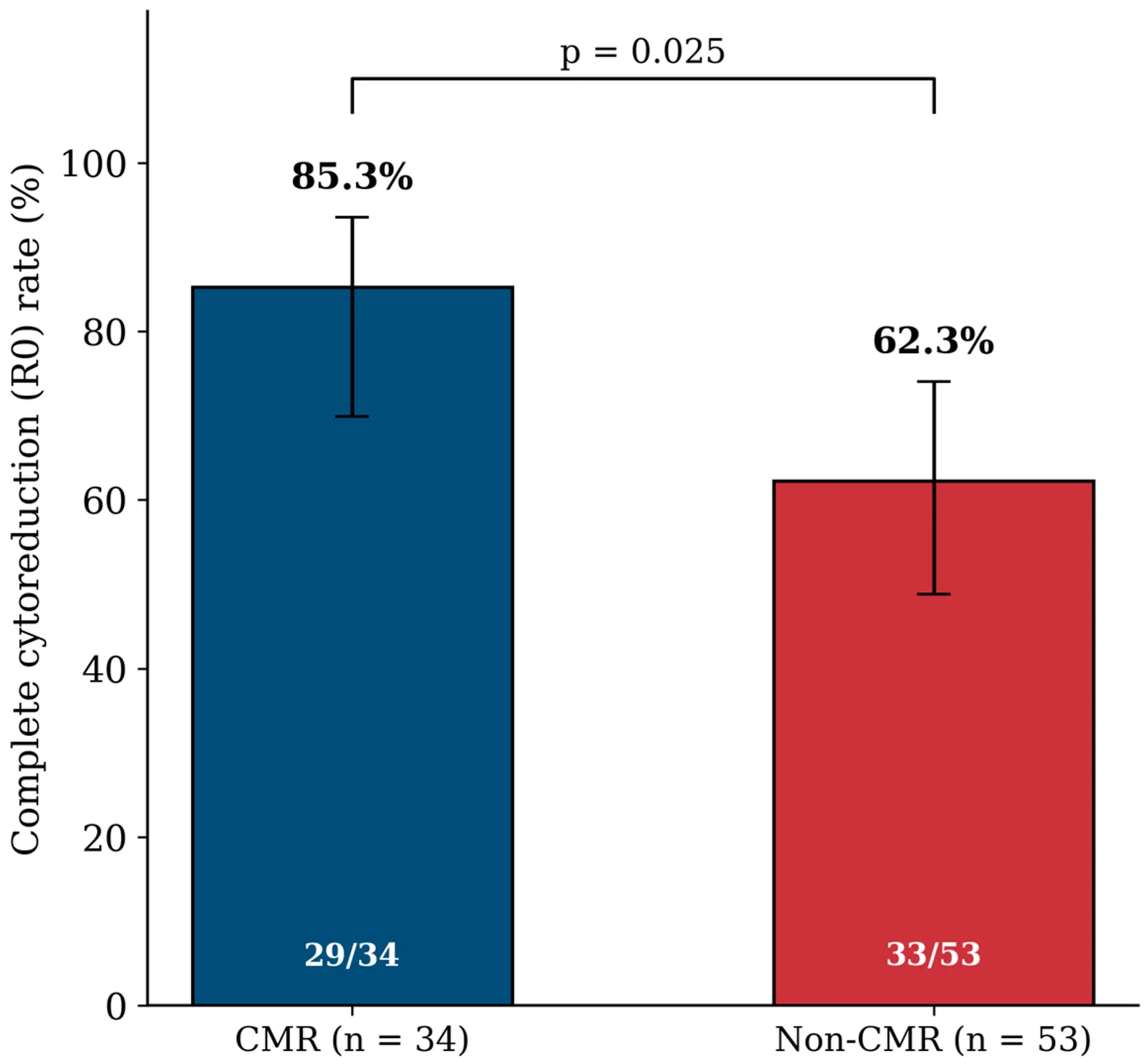
Complete cytoreduction (R0) according to post-NACT metabolic response. Bars show the proportion of patients achieving complete cytoreduction (no macroscopic residual disease) in the complete metabolic response (CMR) and non-CMR groups. Error bars represent 95% confidence intervals.

Complete cytoreduction (R0) was achieved in 62 patients (71.3%). The R0 rate was higher in patients with CMR than in those without CMR (85.3% vs. 62.3%; OR 3.52, 95% CI 1.17–10.56; *p* = 0.025) (Figure 4, Table 2). The sensitivity, specificity, positive predictive value (PPV), and negative predictive value (NPV) of CMR for complete cytoreduction were 46.8% (95% CI 34.9–59.0), 80.0% (60.9–91.1), 85.3% (69.9–93.6), and 37.7% (25.9–51.2), respectively.

**Table 2:** Univariable logistic regression for complete cytoreduction (R0).

Variable	OR (95% CI)	*p*
CMR (yes vs. no)	3.52 (1.17–10.56)	0.025
Age (per year)	0.99 (0.94–1.04)	0.717
FIGO IV (vs. III)	0.82 (0.29–2.27)	0.698
NACT cycles (per cycle)	1.04 (0.71–1.53)	0.833
Post-NACT CA-125 (per log unit)	0.77 (0.53–1.10)	0.155
BMI (per unit)	1.04 (0.95–1.13)	0.368

OR, odds ratio; CI, confidence interval. Other abbreviations as in Table 1. Univariable analyses were exploratory.

In multivariable logistic regression adjusted for age, FIGO stage, and number of NACT cycles, CMR remained independently associated with complete cytoreduction (adjusted OR 3.56, 95% CI 1.18–10.75; *p* = 0.024) (Table 3). This association was preserved after further adjustment for log-transformed post-NACT CA-125 (adjusted OR 3.14, 95% CI 1.00–9.81; *p* = 0.049) and when the number of cycles was analyzed as a categorical variable (adjusted OR 3.81, 95% CI 1.24–11.73; *p* = 0.020).

A CRS 3 was identified in 16 patients (18.4%) and was more frequent in the CMR group than in the non-CMR group (38.2% vs. 5.7%; *p* < 0.001). Complete cytoreduction was achieved in all 16 patients with CRS 3 (100%), compared with 46 of 71 patients (64.8%) with CRS 1–2 (*p* = 0.004).

**Figure 5:**
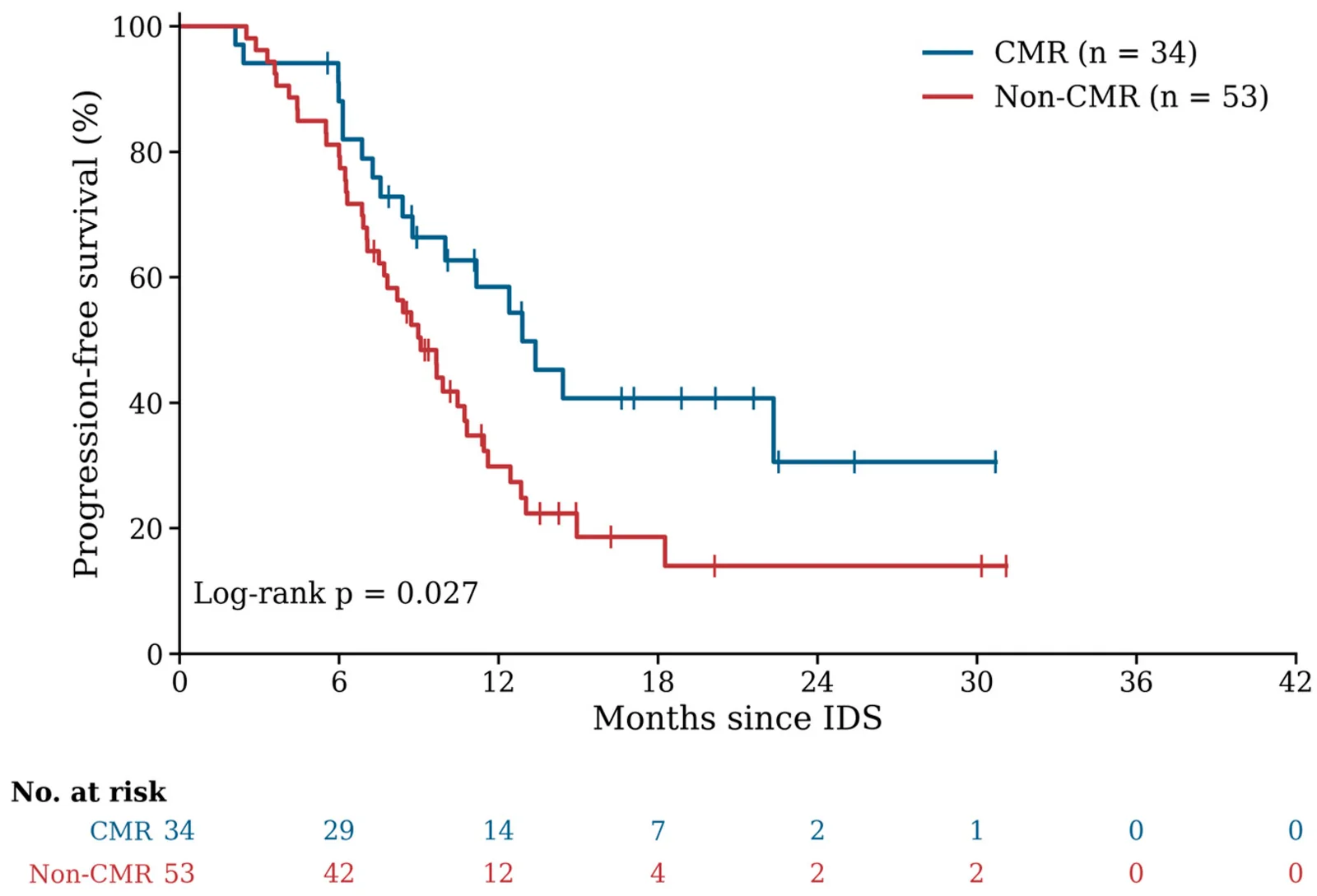
Kaplan–Meier curves of progression-free survival (PFS) according to post-NACT metabolic response, using interval debulking surgery (IDS) as the landmark and time origin. Tick marks indicate censored observations, and the numbers at risk are shown below the plot. CMR, complete metabolic response; IDS, interval debulking surgery.

**Figure 6:**
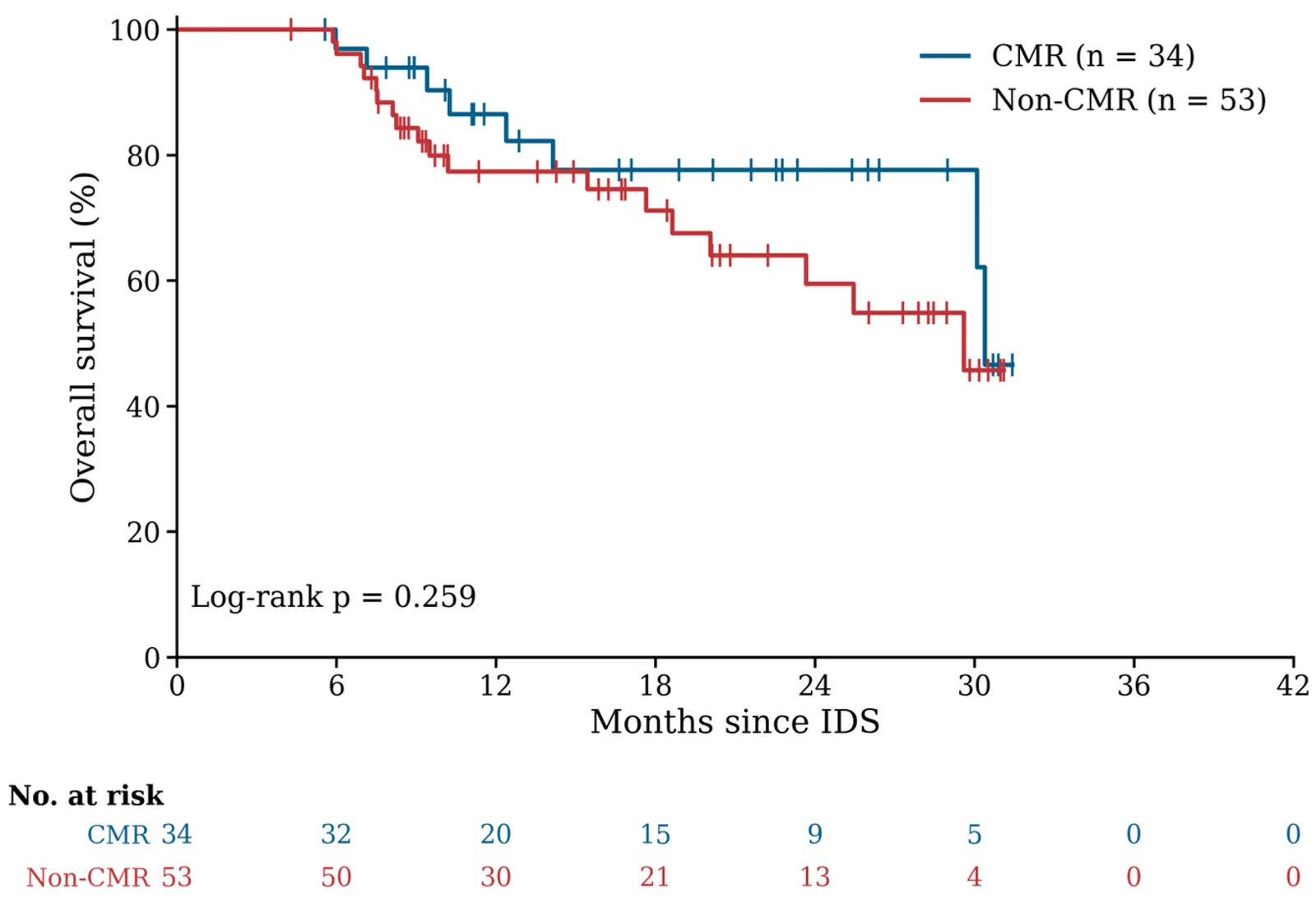
Kaplan–Meier curves of overall survival according to post-NACT metabolic response, using interval debulking surgery (IDS) as the landmark and time origin. Tick marks indicate censored observations, and the numbers at risk are shown below the plot. CMR, complete metabolic response; IDS, interval debulking surgery.

During a median follow-up of 20.2 months from the interval debulking surgery (IDS) landmark (reverse Kaplan–Meier method), 58 patients developed recurrence and 26 died. Median landmark PFS was longer in the CMR group than in the non-CMR group (12.9 vs. 9.1 months; log-rank *p* = 0.027) (Figure 5). The estimated landmark PFS rates at 12 months were 58.5% (95% CI 39.0–73.7) in the CMR group and 29.8% (95% CI 17.5–43.2) in the non-CMR group; the corresponding 24-month PFS rates were 30.6% (95% CI 11.1–52.8) and 14.0% (95% CI 4.7–28.2), respectively. The estimated 24-month OS rates were 77.7% (95% CI 56.3–89.5) in the CMR group and 59.5% (95% CI 40.9–73.9) in the non-CMR group; OS did not differ significantly between the groups (log-rank *p* = 0.259) (Figure 6).

In the IDS-landmark multivariable Cox model adjusted for age, FIGO stage, and number of NACT cycles, CMR was associated with a lower hazard of a PFS event (HR 0.56, 95% CI 0.32–0.99; *p* = 0.046). After further adjustment for complete cytoreduction, the CMR–PFS association was attenuated and no longer statistically significant (HR 0.97, 95% CI 0.52–1.83; *p* = 0.93), whereas complete cytoreduction remained independently associated with PFS (HR 0.20, 95% CI 0.10–0.37; *p* < 0.001) (Table 4). Tied event times were handled using the Breslow method, and the proportional-hazards assumption, assessed with scaled Schoenfeld residuals, showed no evidence of violation.

In summary, post-NACT CMR was associated with complete cytoreduction after adjustment for age, FIGO stage, and the number of NACT cycles. Nevertheless, 33 of 53 patients without CMR (62.3%) also achieved R0. In the IDS-landmark analysis, CMR was associated with longer PFS in Model A, but this association was attenuated and was no longer statistically significant after additional adjustment for R0 status in Model B. No statistically significant difference in OS was observed between the CMR and non-CMR groups.

## 4. Discussion

The principal finding of this study was that post-NACT CMR was independently associated with complete cytoreduction but, because of its limited sensitivity, should not be used as a stand-alone criterion for selecting patients for IDS. In this cohort of patients with advanced high-grade serous tubo-ovarian carcinoma, the association between CMR and complete cytoreduction remained consistent across multivariable and sensitivity analyses. As a preoperative imaging marker, CMR may help identify patients who are more likely to achieve complete cytoreduction.

These findings are consistent with previous studies reporting higher R0 resection rates in patients with favorable metabolic responses. Martoni et al. reported that normalization of FDG uptake after NACT was associated with a high likelihood of R0 resection [9], and an ancillary analysis of the CHIVA trial similarly found that complete cytoreduction was more frequent in metabolic responders than in non-responders [10]. Early sequential FDG-PET during chemotherapy has also been shown to predict treatment response in advanced ovarian cancer [14], and a marked reduction in post-NACT metabolic activity has been associated with histopathological response and PERCIST-based treatment-response evaluation [15,16]. In contrast, Chung et al. found no difference in the R0 rate between the CMR and non-CMR groups [5]. A possible explanation for this discrepancy is the timing of PET/CT assessment. In our cohort, PET/CT was performed after completion of NACT and immediately before surgery, whereas Chung et al. assessed metabolic response after three cycles of NACT, despite some patients subsequently receiving a fourth cycle before IDS. Consequently, PET/CT findings in their study may not have reflected the tumor burden immediately before surgery as closely as in our cohort. The association between CMR and CRS 3 further supports the biological plausibility of our findings and suggests concordance between metabolic and pathological responses to chemotherapy [17]. However, the observational design of this study does not allow us to establish the mechanisms underlying the association between CMR and complete cytoreduction.

In the IDS-landmark analysis, median PFS was longer in the CMR group than in the non-CMR group (12.9 versus 9.1 months). CMR was associated with PFS after adjustment for age, FIGO stage, and the number of NACT cycles, but the association was attenuated and was no longer statistically significant after additional adjustment for R0 status. This attenuation is compatible with overlapping prognostic information between CMR and R0 but does not establish that R0 mediates the association between CMR and PFS. No statistically significant difference in OS was observed. Given the limited number of deaths and follow-up duration, the OS findings remain uncertain and should not be interpreted as evidence of equivalent survival.

From a clinical perspective, CMR had a PPV of 85.3% for complete cytoreduction; however, its sensitivity was low, and R0 resection was still achieved in more than half of the patients without CMR. Because predictive values depend on the underlying rate of complete cytoreduction, these estimates are specific to this selected single-center IDS cohort and may vary across populations with different R0 rates. Two distinct issues should be distinguished: the prediction of complete cytoreduction and the detection of residual disease. First, a substantial proportion of patients without CMR still achieved R0, because residual FDG-avid disease is frequently still amenable to complete surgical resection; persistent metabolic activity therefore does not necessarily indicate unresectable disease, and the absence of CMR does not preclude complete cytoreduction. Second, and separately, PET/CT has limited sensitivity for detecting small-volume residual disease—such as miliary peritoneal implants, low-volume serosal spread, and occult nodal metastases—which may fall below its spatial resolution or be obscured by physiological or inflammatory uptake [18]; this concerns the ability of PET/CT to exclude residual disease rather than the resectability of metabolically active lesions. The absence of CMR alone should not be used to exclude patients from IDS. This caution is reinforced by Lee et al., who reported that PET/CT did not outperform contrast-enhanced CT for detecting residual disease after NACT and could miss disease at certain anatomical sites [19]. Metabolic response may therefore be most useful for identifying patients with a greater probability of achieving complete cytoreduction, rather than for excluding patients from IDS.

The strengths of this study include a histologically uniform cohort of high-grade serous carcinoma and a single-center design with consistent surgical and pathological assessment. All PET/CT examinations were acquired on a single scanner and re-evaluated specifically for the study by two experienced nuclear medicine physicians, each with more than 10 years of experience, who were blinded to the surgical and pathological findings, with disagreements resolved by consensus; this provided a standardized assessment of metabolic response rather than reliance on heterogeneous clinical reports. The analysis enabled the concurrent evaluation of metabolic response, surgical outcomes, and survival, and the primary multivariable model was complemented by sensitivity analyses and formal assessment of the proportional hazards assumption, which were performed to assess the consistency of the findings.

This study has several limitations. Its retrospective, single-center design and modest sample size limit the precision of the estimates and the generalizability of the findings; in particular, only 25 patients had residual disease, which reduced the precision of the multivariable logistic regression and produced wide confidence intervals, so the magnitude of the CMR–complete-cytoreduction association should be interpreted with caution. Metabolic response was assessed visually against hepatic activity by two independent readers blinded to the surgical and histopathological findings, with disagreements resolved by consensus, but without formal SULpeak or volumetric (metabolic tumor volume or total lesion glycolysis) quantification. BRCA mutation and homologous recombination deficiency (HRD) status and post-operative maintenance therapy (e.g., PARP inhibitors, bevacizumab) were not systematically recorded and may act as unmeasured confounders, particularly for the survival associations; unmeasured genetic status may also confound the association between CMR and complete cytoreduction. In addition, standardized measures of disease burden, laparoscopic resectability, and surgical complexity were unavailable, which limits further adjustment of this association and is not fully addressed by reporting organ-resection counts.

Because the cohort included only patients who proceeded to IDS, the findings may not be generalizable to patients who were not selected for surgery after NACT, and the study did not include a comparison group managed without PET/CT, so it cannot establish the incremental value of PET/CT for IDS selection. As the multidisciplinary tumor board had access to the routine clinical post-NACT PET/CT, the treating surgeons were not blinded to these findings, which may have influenced patient selection and surgical planning; the extent to which these findings influenced surgical planning or targeted resection was not quantified. The limited follow-up and number of deaths (*n* = 26) reduced the precision of the OS assessment; no multivariable Cox model was fitted for overall survival because of the risk of overfitting, and an adjusted CMR–OS association could therefore not be evaluated. To mitigate guarantee-time (immortal-time) bias, the survival analyses used the date of IDS as the time origin (landmark analysis); however, this removes only the pre-IDS interval from the analysis and does not eliminate the selection bias arising from restricting the cohort to patients who reached IDS, nor residual confounding by unmeasured factors.

Future studies should be designed as prospective, multicenter investigations with standardized image acquisition and independent, blinded readers. Incorporating quantitative and volumetric PET/CT parameters, such as SULpeak, metabolic tumor volume, and total lesion glycolysis, may further refine the assessment of metabolic response and its association with complete cytoreduction. Among these, total lesion glycolysis has been reported to predict outcome after NACT in epithelial ovarian cancer [20]. Radiomic and artificial intelligence-based analyses of PET/CT may further improve the preoperative prediction of resectability. External validation in independent cohorts is warranted before CMR is used to inform preoperative decision-making.

## 5. Conclusions

In patients with advanced high-grade serous tubo-ovarian carcinoma, CMR on post-NACT 18F-FDG PET/CT was independently associated with complete cytoreduction. However, because of its limited sensitivity, the absence of CMR alone should not preclude IDS. CMR may therefore serve as an adjunctive imaging biomarker for estimating the likelihood of complete cytoreduction before IDS. Validation in larger multicenter cohorts is warranted to determine whether CMR can be integrated into preoperative decision-making algorithms for IDS.

## Figures and Tables

**Table 3:** Multivariable logistic regression for complete cytoreduction (R0).

Variable	Adjusted OR (95% CI)	*p*
CMR (yes vs. no)	3.56 (1.18–10.75)	0.024
Age (per year)	0.99 (0.94–1.05)	0.791
FIGO IV (vs. III)	0.79 (0.27–2.32)	0.674
NACT cycles (per cycle)	1.09 (0.73–1.64)	0.674

The primary multivariable model was adjusted for age, FIGO stage, and number of NACT cycles. In sensitivity analyses, the adjusted OR for CMR was 3.14 (1.00–9.81; *p* = 0.049) after further adjustment for log-transformed post-NACT CA-125, and 3.81 (1.24–11.73; *p* = 0.020) when the number of NACT cycles was modeled as a categorical variable. Abbreviations as in Table 1 and Table 2.

**Table 4:** Multivariable Cox proportional hazards models for progression-free survival.

Model	Variable	HR (95% CI)	*p*
Model A	CMR (yes vs. no)	0.56 (0.32–0.99)	0.046
	Age (per year)	1.00 (0.97–1.02)	0.747
	FIGO IV (vs. III)	1.26 (0.70–2.28)	0.444
	NACT cycles (per cycle)	1.04 (0.84–1.29)	0.706
Model B	CMR (yes vs. no)	0.97 (0.52–1.83)	0.93
	Age (per year)	0.99 (0.96–1.02)	0.355
	FIGO IV (vs. III)	1.13 (0.63–2.04)	0.684
	NACT cycles (per cycle)	1.14 (0.91–1.43)	0.241
	Complete cytoreduction (R0)	0.20 (0.10–0.37)	<0.001

Model A included CMR, age, FIGO stage, and number of NACT cycles; Model B additionally included complete cytoreduction. HR, hazard ratio. Other abbreviations as in Table 1 and Table 2.

## Data Availability

The data presented in this study are available on request from the corresponding author due to privacy and ethical reasons.

## Abbreviations

The following abbreviations are used in this manuscript:

CMR	complete metabolic response
NACT	neoadjuvant chemotherapy
IDS	interval debulking surgery
PET/CT	positron emission tomography/computed tomography
18F-FDG	18F-fluorodeoxyglucose
R0	no macroscopic residual disease (complete cytoreduction)
PFS	progression-free survival
OS	overall survival
CRS	chemotherapy response score
FIGO	International Federation of Gynecology and Obstetrics
CA-125	cancer antigen 125
PERCIST	PET Response Criteria in Solid Tumors
RECIST	Response Evaluation Criteria in Solid Tumors
SUV	standardized uptake value
MTV	metabolic tumor volume
TLG	total lesion glycolysis
CT	computed tomography
OR	odds ratio
HR	hazard ratio
CI	confidence interval
IQR	interquartile range
PPV	positive predictive value
NPV	negative predictive value
BMI	body mass index
